# 1,2-Diphenyl­ethane-1,2-diyl diiso­nico­tinate monohydrate^1^

**DOI:** 10.1107/S1600536808022484

**Published:** 2008-09-24

**Authors:** Jiang Yan, Meng Qi, Xi Haitao, Sun Xiaoqiang, Wang Xin

**Affiliations:** a Materials Chemistry Laboratory, Nanjing University of Science and Technology, Nanjing 210094, People’s Republic of China; b School of Chemistry and Chemical Engineering, Jiangsu Polytechnic University, Changzhou 213164, People’s Republic of China

## Abstract

In the novel title compound, C_26_H_20_N_2_O_4_·H_2_O, the two phenyl rings make a dihedral angle of 45.3 (1)° with each other, and the dihedral angle between the two pyridyl planes is 69.8 (1)°.

## Related literature

For  general background, see: Aspinall *et al.* (2003 ▶); Takenaka *et al.* (2006 ▶); MacMahon *et al.* (2001 ▶); Schuster *et al.* (2005 ▶). For related structures, see: Shi *et al.* (2006 ▶).
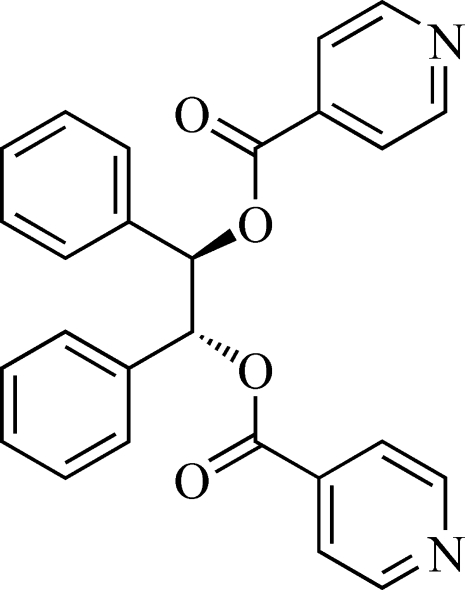


## Experimental

### 

#### Crystal data

C_26_H_20_N_2_O_4_·H_2_O
*M* *_r_* = 442.46
Monoclinic, 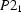

*a* = 11.925 (10) Å
*b* = 5.826 (5) Å
*c* = 17.787 (15) Å
β = 105.629 (10)°
*V* = 1190.1 (17) Å^3^
*Z* = 2
Mo *K*α radiation
μ = 0.09 mm^−1^
*T* = 291 (2) K
0.32 × 0.24 × 0.22 mm

#### Data collection

Bruker SMART APEX CCD diffractometer
Absorption correction: multi-scan (*SADABS*; Bruker,2000 ▶) *T*_min_ = 0.979, *T*_max_ = 0.981
6535 measured reflections
2566 independent reflections
1816 reflections with *I* > 2σ(*I*)
*R*_int_ = 0.024

#### Refinement

*R*[*F* ^2^ > 2σ(*F* ^2^)] = 0.046
*wR*(*F* ^2^) = 0.086
*S* = 1.10
2566 reflections
304 parameters
1 restraint
H atoms treated by a mixture of independent and constrained refinement
Δρ_max_ = 0.13 e Å^−3^
Δρ_min_ = −0.11 e Å^−3^

### 

Data collection: *SMART* (Bruker, 2000 ▶); cell refinement: *SAINT* (Bruker, 2000 ▶); data reduction: *SAINT*; program(s) used to solve structure: *SHELXTL* (Sheldrick, 2008 ▶); program(s) used to refine structure: *SHELXTL*; molecular graphics: *SHELXTL*; software used to prepare material for publication: *SHELXTL*.

## Supplementary Material

Crystal structure: contains datablocks z0612, I. DOI: 10.1107/S1600536808022484/sg2251sup1.cif

Structure factors: contains datablocks I. DOI: 10.1107/S1600536808022484/sg2251Isup2.hkl

Additional supplementary materials:  crystallographic information; 3D view; checkCIF report

## Figures and Tables

**Table 1:** Hydrogen-bond geometry (Å, °)

*D*—H⋯*A*	*D*—H	H⋯*A*	*D*⋯*A*	*D*—H⋯*A*
O5—H5*B*⋯N2^i^	0.84 (4)	2.48 (5)	2.921 (4)	113 (4)

Symmetry code: (i) 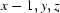.

**Fig. 1.**
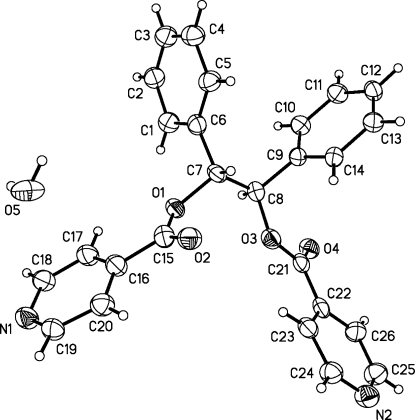
The molecular structure of (I), showing 20% probability displacement ellipsoids.

**Fig. 2.**
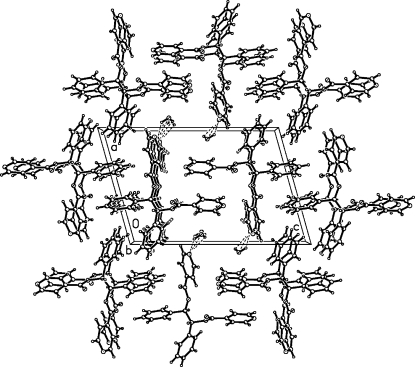
The molecular packing diagram in the crystal for (I).

## supplementary crystallographic information

### Comment

Synthesis of chiral molecules containing pyridine rings has attracted considerable attention in recent years (Aspinall,*et al.*, 2003; Takenaka,*et al.*, 2006; MacMahon, *et al.*, 2001; Schuster, *et al.*, 2005). In the  title compound, (I), C_26_H_20_NO_2_, all bond lengths and angles show normal values (Shi, *et al.*, 2006). The chiral molecule (Figure 1) consists of two benzene rings and two pyridine rings. The dihedral angle between the two benzene ring is 45.28°. The torsion angle C2—C1—C7—O2 is -44.2 (2)°; the torsional angle  The packing arrangement in a unit cell of the title molecule is shown in Figure 2.

### Experimental

The title compound, was synthesized by the reaction of *trans*-1,2-stilbene with nicotinic acid in dichloromethane. The single crystals of (I) suitable for X-ray diffraction were obtained from an ethanol solution by slow evaporation.

### Refinement

The H atoms bonded to N atom were located from difference density maps and refined isotropically. The H atoms bonded to C atoms were located geometrically and treated as riding, with C—H distances of 0.95–1.00 Å and with *U*_iso_(H) = 1.5Ueq(C) for methyl H atoms and 1.2Ueq(C) for others. Due to the absence of heavy atoms corresponding to Si, it was impossible to determine the absolute configuration in this case. At this stage, the Friedel pairs were merged.

### Crystal data

**Table:**

C_26_H_20_N_2_O_4_·H_2_O	*F*(000) = 464
*M**_r_* = 442.46	*D*_x_ = 1.235 Mg m^−^^3^
Monoclinic, *P*2_1_	Melting point: 327 K
Hall symbol: P 2yb	Mo *K*α radiation, λ = 0.71073 Å
*a* = 11.925 (10) Å	Cell parameters from 1142 reflections
*b* = 5.826 (5) Å	θ = 2.4–21.6°
*c* = 17.787 (15) Å	µ = 0.09 mm^−^^1^
β = 105.629 (10)°	*T* = 291 K
*V* = 1190.1 (17) Å^3^	Acicular, colorless
*Z* = 2	0.32 × 0.24 × 0.22 mm

### Data collection

**Table:**

Bruker SMART APEX CCD diffractometer	2566 independent reflections
Radiation source: sealed tube	1816 reflections with *I* > 2σ(*I*)
graphite	*R*_int_ = 0.024
phi and ω scans	θ_max_ = 26.0°, θ_min_ = 1.8°
Absorption correction: multi-scan (SADABS; Bruker,2000)	*h* = −14→14
*T*_min_ = 0.979, *T*_max_ = 0.981	*k* = −7→7
6535 measured reflections	*l* = −21→18

### Refinement

**Table:**

Refinement on *F*^2^	Primary atom site location: structure-invariant direct methods
Least-squares matrix: full	Secondary atom site location: difference Fourier map
*R*[*F*^2^ > 2σ(*F*^2^)] = 0.046	Hydrogen site location: inferred from neighbouring sites
*wR*(*F*^2^) = 0.086	H atoms treated by a mixture of independent and constrained refinement
*S* = 1.10	*w* = 1/[σ^2^(*F*_o_^2^) + (0.030*P*)^2^] where *P* = (*F*_o_^2^ + 2*F*_c_^2^)/3
2566 reflections	(Δ/σ)_max_ < 0.001
304 parameters	Δρ_max_ = 0.13 e Å^−^^3^
1 restraint	Δρ_min_ = −0.11 e Å^−^^3^

### Special details

**Table:**

Geometry. All esds (except the esd in the dihedral angle between two l.s. planes) are estimated using the full covariance matrix. The cell esds are taken into account individually in the estimation of esds in distances, angles and torsion angles; correlations between esds in cell parameters are only used when they are defined by crystal symmetry. An approximate (isotropic) treatment of cell esds is used for estimating esds involving l.s. planes.
Refinement. Refinement of *F*^2^ against ALL reflections. The weighted *R*-factor *wR* and goodness of fit *S* are based on *F*^2^, conventional *R*-factors *R* are based on *F*, with *F* set to zero for negative *F*^2^. The threshold expression of *F*^2^ > σ(*F*^2^) is used only for calculating *R*-factors(gt) *etc*. and is not relevant to the choice of reflections for refinement. *R*-factors based on *F*^2^ are statistically about twice as large as those based on *F*, and *R*- factors based on ALL data will be even larger.

### Fractional atomic coordinates and isotropic or equivalent isotropic displacement parameters (Å^2^)

**Table:**

	*x*	*y*	*z*	*U*_iso_*/*U*_eq_	
C1	0.1366 (3)	0.0950 (8)	0.1997 (2)	0.0617 (9)	
H1	0.1843	0.0198	0.2426	0.074*	
C2	0.0250 (3)	0.0040 (7)	0.1606 (2)	0.0610 (10)	
H2	0.0002	−0.1319	0.1784	0.073*	
C3	−0.0446 (3)	0.1107 (7)	0.0988 (2)	0.0658 (10)	
H3	−0.1171	0.0486	0.0744	0.079*	
C4	−0.0107 (3)	0.3060 (7)	0.0718 (2)	0.0644 (10)	
H4	−0.0598	0.3783	0.0287	0.077*	
C5	0.1006 (3)	0.4042 (7)	0.1088 (2)	0.0604 (9)	
H5	0.1237	0.5395	0.0895	0.072*	
C6	0.1729 (3)	0.2994 (6)	0.17230 (19)	0.0526 (8)	
C7	0.2931 (2)	0.3938 (6)	0.20841 (16)	0.0479 (8)	
H7	0.2980	0.5524	0.1912	0.058*	
C8	0.3874 (3)	0.2465 (7)	0.18741 (17)	0.0520 (8)	
H8	0.3854	0.0892	0.2065	0.062*	
C9	0.3699 (2)	0.2474 (6)	0.10007 (17)	0.0454 (7)	
C10	0.3172 (3)	0.0645 (7)	0.05519 (19)	0.0575 (9)	
H10	0.2938	−0.0621	0.0790	0.069*	
C11	0.2988 (3)	0.0677 (7)	−0.0256 (2)	0.0592 (9)	
H11	0.2647	−0.0576	−0.0555	0.071*	
C12	0.3315 (3)	0.2587 (7)	−0.06095 (19)	0.0545 (8)	
H12	0.3165	0.2639	−0.1150	0.065*	
C13	0.3855 (3)	0.4387 (7)	−0.01715 (19)	0.0560 (9)	
H13	0.4093	0.5640	−0.0414	0.067*	
C14	0.4059 (2)	0.4372 (6)	0.06493 (19)	0.0511 (8)	
H14	0.4427	0.5604	0.0949	0.061*	
C15	0.3427 (3)	0.5843 (7)	0.3341 (2)	0.0534 (8)	
C16	0.3422 (3)	0.5479 (7)	0.4175 (2)	0.0586 (9)	
C17	0.2955 (3)	0.3621 (7)	0.4423 (2)	0.0629 (10)	
H17	0.2644	0.2469	0.4066	0.076*	
C18	0.2919 (3)	0.3359 (7)	0.5200 (2)	0.0611 (10)	
H18	0.2595	0.2051	0.5356	0.073*	
C19	0.3806 (3)	0.6880 (6)	0.5480 (2)	0.0611 (10)	
H19	0.4073	0.8048	0.5840	0.073*	
C20	0.3910 (3)	0.7229 (7)	0.4693 (2)	0.0639 (10)	
H20	0.4270	0.8507	0.4548	0.077*	
C21	0.5955 (3)	0.2419 (6)	0.22411 (18)	0.0478 (8)	
C22	0.7001 (3)	0.3845 (6)	0.25533 (17)	0.0504 (8)	
C23	0.7001 (3)	0.5913 (7)	0.29022 (18)	0.0551 (9)	
H23	0.6312	0.6513	0.2971	0.066*	
C24	0.8028 (3)	0.7121 (7)	0.3155 (2)	0.0608 (10)	
H24	0.8009	0.8528	0.3398	0.073*	
C25	0.9004 (3)	0.4385 (8)	0.2745 (2)	0.0661 (10)	
H25	0.9706	0.3818	0.2690	0.079*	
C26	0.8048 (3)	0.2999 (7)	0.2472 (2)	0.0590 (9)	
H26	0.8101	0.1579	0.2245	0.071*	
N1	0.3370 (3)	0.5066 (6)	0.57225 (18)	0.0654 (8)	
N2	0.9046 (3)	0.6389 (6)	0.30715 (18)	0.0660 (9)	
O1	0.31042 (17)	0.3875 (4)	0.29344 (11)	0.0516 (6)	
O2	0.36767 (19)	0.7536 (5)	0.30737 (14)	0.0629 (7)	
O3	0.49642 (18)	0.3587 (4)	0.22757 (12)	0.0531 (6)	
O4	0.59297 (19)	0.0481 (5)	0.19752 (13)	0.0587 (6)	
O5	0.0848 (2)	0.9233 (6)	0.40864 (18)	0.0779 (9)	
H5A	0.059 (4)	0.815 (9)	0.424 (3)	0.093*	
H5B	0.047 (3)	0.947 (8)	0.362 (3)	0.093*	

### Atomic displacement parameters (Å^2^)

**Table:**

	*U*^11^	*U*^22^	*U*^33^	*U*^12^	*U*^13^	*U*^23^
C1	0.061 (2)	0.065 (3)	0.058 (2)	0.0020 (18)	0.0136 (18)	0.0043 (19)
C2	0.063 (2)	0.059 (3)	0.061 (2)	−0.0087 (17)	0.0178 (19)	−0.0038 (18)
C3	0.062 (2)	0.069 (3)	0.064 (2)	−0.010 (2)	0.0140 (19)	−0.015 (2)
C4	0.060 (2)	0.067 (3)	0.062 (2)	0.0080 (19)	0.0089 (18)	−0.005 (2)
C5	0.0522 (18)	0.063 (2)	0.064 (2)	0.0058 (18)	0.0123 (16)	−0.001 (2)
C6	0.0556 (19)	0.057 (2)	0.0475 (18)	−0.0030 (16)	0.0178 (15)	−0.0043 (17)
C7	0.0533 (17)	0.057 (2)	0.0308 (15)	0.0098 (16)	0.0067 (13)	0.0048 (15)
C8	0.0542 (18)	0.062 (2)	0.0420 (17)	−0.0031 (16)	0.0175 (15)	0.0039 (16)
C9	0.0432 (15)	0.053 (2)	0.0411 (18)	0.0037 (15)	0.0136 (14)	−0.0001 (16)
C10	0.0568 (19)	0.065 (2)	0.050 (2)	−0.0001 (18)	0.0130 (16)	−0.0030 (19)
C11	0.061 (2)	0.061 (2)	0.054 (2)	−0.0055 (19)	0.0127 (17)	−0.0121 (19)
C12	0.0562 (18)	0.067 (2)	0.0429 (19)	0.0085 (18)	0.0180 (16)	0.0023 (18)
C13	0.0502 (17)	0.064 (2)	0.0525 (19)	0.0065 (17)	0.0121 (15)	0.0081 (18)
C14	0.0488 (17)	0.059 (2)	0.0481 (18)	−0.0065 (17)	0.0179 (14)	−0.0004 (17)
C15	0.0568 (19)	0.052 (2)	0.053 (2)	0.0120 (17)	0.0188 (16)	0.0071 (19)
C16	0.064 (2)	0.062 (2)	0.0490 (18)	−0.0077 (18)	0.0140 (16)	−0.0006 (18)
C17	0.066 (2)	0.066 (3)	0.055 (2)	−0.0181 (18)	0.0140 (18)	−0.0070 (19)
C18	0.067 (2)	0.059 (2)	0.055 (2)	−0.0162 (18)	0.0121 (17)	0.0051 (18)
C19	0.065 (2)	0.060 (2)	0.057 (2)	−0.0133 (19)	0.0159 (17)	−0.0154 (19)
C20	0.0620 (19)	0.063 (2)	0.065 (2)	−0.0051 (18)	0.0138 (18)	−0.0064 (19)
C21	0.0522 (17)	0.058 (2)	0.0351 (16)	0.0071 (16)	0.0142 (14)	0.0124 (16)
C22	0.0616 (19)	0.060 (2)	0.0320 (15)	0.0033 (17)	0.0170 (14)	0.0072 (16)
C23	0.060 (2)	0.065 (3)	0.0394 (17)	0.0064 (17)	0.0124 (15)	0.0017 (17)
C24	0.060 (2)	0.063 (3)	0.058 (2)	0.0028 (18)	0.0136 (18)	−0.0088 (18)
C25	0.063 (2)	0.064 (3)	0.072 (2)	−0.001 (2)	0.0174 (19)	−0.011 (2)
C26	0.0579 (19)	0.061 (2)	0.058 (2)	−0.0075 (17)	0.0152 (17)	−0.0077 (18)
N1	0.0718 (19)	0.066 (2)	0.0574 (17)	−0.0158 (16)	0.0152 (15)	−0.0097 (16)
N2	0.0662 (18)	0.066 (2)	0.065 (2)	0.0032 (15)	0.0164 (15)	−0.0170 (17)
O1	0.0563 (13)	0.0607 (16)	0.0372 (12)	−0.0064 (12)	0.0117 (10)	0.0014 (11)
O2	0.0610 (14)	0.0649 (17)	0.0592 (15)	−0.0069 (13)	0.0100 (12)	0.0042 (14)
O3	0.0570 (12)	0.0594 (15)	0.0429 (11)	0.0088 (11)	0.0134 (10)	−0.0027 (11)
O4	0.0579 (13)	0.0690 (17)	0.0498 (13)	0.0184 (13)	0.0153 (10)	−0.0072 (13)
O5	0.0623 (17)	0.073 (2)	0.079 (2)	−0.0134 (15)	−0.0127 (14)	0.0160 (17)

### Geometric parameters (Å, °)

**Table:**

C1—C6	1.399 (5)	C15—O2	1.168 (4)
C1—C2	1.427 (5)	C15—O1	1.354 (4)
C1—H1	0.9300	C15—C16	1.502 (5)
C2—C3	1.339 (5)	C16—C17	1.344 (5)
C2—H2	0.9300	C16—C20	1.392 (5)
C3—C4	1.339 (6)	C17—C18	1.402 (5)
C3—H3	0.9300	C17—H17	0.9300
C4—C5	1.433 (5)	C18—N1	1.369 (5)
C4—H4	0.9300	C18—H18	0.9300
C5—C6	1.367 (5)	C19—N1	1.302 (5)
C5—H5	0.9300	C19—C20	1.452 (5)
C6—C7	1.507 (4)	C19—H19	0.9300
C7—O1	1.471 (3)	C20—H20	0.9300
C7—C8	1.538 (4)	C21—O4	1.221 (5)
C7—H7	0.9800	C21—O3	1.379 (4)
C8—O3	1.459 (4)	C21—C22	1.477 (5)
C8—C9	1.511 (4)	C22—C23	1.355 (5)
C8—H8	0.9800	C22—C26	1.386 (4)
C9—C10	1.377 (5)	C23—C24	1.379 (5)
C9—C14	1.394 (5)	C23—H23	0.9300
C10—C11	1.394 (5)	C24—N2	1.333 (4)
C10—H10	0.9300	C24—H24	0.9300
C11—C12	1.385 (5)	C25—N2	1.298 (5)
C11—H11	0.9300	C25—C26	1.374 (5)
C12—C13	1.361 (5)	C25—H25	0.9300
C12—H12	0.9300	C26—H26	0.9300
C13—C14	1.414 (5)	O5—H5A	0.78 (5)
C13—H13	0.9300	O5—H5B	0.84 (4)
C14—H14	0.9300		
			
C6—C1—C2	118.9 (3)	C9—C14—C13	118.7 (3)
C6—C1—H1	120.6	C9—C14—H14	120.7
C2—C1—H1	120.6	C13—C14—H14	120.7
C3—C2—C1	121.2 (4)	O2—C15—O1	124.4 (3)
C3—C2—H2	119.4	O2—C15—C16	126.2 (4)
C1—C2—H2	119.4	O1—C15—C16	109.4 (3)
C4—C3—C2	120.6 (4)	C17—C16—C20	120.7 (3)
C4—C3—H3	119.7	C17—C16—C15	123.3 (3)
C2—C3—H3	119.7	C20—C16—C15	116.0 (4)
C3—C4—C5	120.4 (4)	C16—C17—C18	122.4 (3)
C3—C4—H4	119.8	C16—C17—H17	118.8
C5—C4—H4	119.8	C18—C17—H17	118.8
C6—C5—C4	120.1 (4)	N1—C18—C17	118.6 (3)
C6—C5—H5	119.9	N1—C18—H18	120.7
C4—C5—H5	119.9	C17—C18—H18	120.7
C5—C6—C1	118.8 (3)	N1—C19—C20	125.3 (3)
C5—C6—C7	120.3 (3)	N1—C19—H19	117.3
C1—C6—C7	120.7 (3)	C20—C19—H19	117.3
O1—C7—C6	106.5 (2)	C16—C20—C19	113.9 (4)
O1—C7—C8	109.0 (2)	C16—C20—H20	123.0
C6—C7—C8	111.5 (3)	C19—C20—H20	123.0
O1—C7—H7	109.9	O4—C21—O3	122.8 (3)
C6—C7—H7	109.9	O4—C21—C22	126.6 (3)
C8—C7—H7	109.9	O3—C21—C22	110.6 (3)
O3—C8—C9	111.1 (2)	C23—C22—C26	118.4 (3)
O3—C8—C7	104.2 (3)	C23—C22—C21	124.6 (3)
C9—C8—C7	109.9 (3)	C26—C22—C21	117.0 (3)
O3—C8—H8	110.5	C22—C23—C24	119.6 (3)
C9—C8—H8	110.5	C22—C23—H23	120.2
C7—C8—H8	110.5	C24—C23—H23	120.2
C10—C9—C14	120.1 (3)	N2—C24—C23	123.8 (4)
C10—C9—C8	120.6 (3)	N2—C24—H24	118.1
C14—C9—C8	119.3 (3)	C23—C24—H24	118.1
C9—C10—C11	120.5 (4)	N2—C25—C26	128.0 (4)
C9—C10—H10	119.7	N2—C25—H25	116.0
C11—C10—H10	119.7	C26—C25—H25	116.0
C12—C11—C10	119.6 (3)	C25—C26—C22	116.0 (3)
C12—C11—H11	120.2	C25—C26—H26	122.0
C10—C11—H11	120.2	C22—C26—H26	122.0
C13—C12—C11	120.5 (3)	C19—N1—C18	118.9 (3)
C13—C12—H12	119.8	C25—N2—C24	114.2 (4)
C11—C12—H12	119.8	C15—O1—C7	117.9 (3)
C12—C13—C14	120.6 (3)	C21—O3—C8	114.7 (3)
C12—C13—H13	119.7	H5A—O5—H5B	108 (4)
C14—C13—H13	119.7		

### Hydrogen-bond geometry (Å, °)

**Table:**

*D*—H···*A*	*D*—H	H···*A*	*D*···*A*	*D*—H···*A*
O5—H5B···N2^i^	0.84 (4)	2.48 (5)	2.921 (4)	113 (4)

Symmetry codes: (i) *x*−1, *y*, *z*.
